# The *Mugil curema* species complex (Pisces, Mugilidae): a new karyotype for the Pacific white mullet mitochondrial lineage

**DOI:** 10.3897/CompCytogen.v11i2.11579

**Published:** 2017-04-07

**Authors:** Mauro Nirchio, Claudio Oliveira, Zoila R. Siccha-Ramirez, Viviani F. de Sene, Luciana Sola, Valentina Milana, Anna Rita Rossi

**Affiliations:** 1 Escuela de Ciencias Aplicadas del Mar, Núcleo de Nueva Esparta, Universidad de Oriente, Apartado 174, Porlamar, Isla de Margarita, Venezuela; 2 Universidad Técnica de Machala, Av. Panamericana km 5½, Via Pasaje, Machala, El Oro, Ecuador; 3 Departamento de Morfologia, Instituto de Biociências Universidade Estadual Paulista, 18618-970 Botucatu, São Paulo, Brazil; 4 Dipartimento di Biologia e Biotecnologie “C. Darwin”, Sapienza - Università di Roma, Via Alfonso Borelli 50, 00161, Rome, Italy

**Keywords:** Fish, Mugilidae, cytochrome oxidase subunit I, cytotaxonomy, molecular systematics

## Abstract

Recent molecular phylogenetic analyses have shown that the *Mugil curema* Valenciennes, 1836 species complex includes *M. incilis* Hancock, 1830, *M. thoburni* (Jordan & Starks, 1896) and at least four “*M. curema*” mitochondrial lineages, considered as cryptic species. The cytogenetic data on some representatives of the species complex have shown a high cytogenetic diversity. This research reports the results of cytogenetic and molecular analyses of white mullet collected in Ecuador. The analyzed specimens were molecularly assigned to the *Mugil* sp. O, the putative cryptic species present in the Pacific Ocean and showed a 2n = 46 karyotype, which is composed of 2 metacentric and 44 subtelocentric/acrocentric chromosomes. This karyotype is different from the one described for *M. incilis* (2n = 48) and from those of the two western Atlantic lineages *Mugil curema* (2n = 28), and *Mugil margaritae* (2n = 24). Data suggest the need for a morphological analysis to assign a species name to this Pacific lineage.

## Introduction

The family Mugilidae currently comprises 20 genera and 74 species (Eschmeyer and Fong 2016), which are widely distributed in various tropical, subtropical and temperate coastal regions of the world (Thomson 1997, Froese and Pauly 2016). These fishes show highly conserved morphological and anatomical characteristics, which are often associated with wide distribution ranges and, thus, the family has undergone many taxonomic revisions, both at the genus and species levels (Thomson 1997).

In the last decade, molecular phylogenetic and phylogeographic analyses have revealed that the morphological features commonly used to identify species seem to be insufficient, both to describe the great diversity of species within Mugilidae and to infer the phylogenetic relationships among the species (Durand et al. 2012, Durand and Borsa 2015). More specifically, Durand et al. (2012) showed that a proportion of the species with large distribution ranges, such as *Mugil cephalus* Linnaeus, 1758 and *M. curema* Valenciennes, 1836 consists of cryptic species. Specifically referring to *M. curema*, different mtDNA lineages had been previously identified along the American Atlantic coasts by Heras et al. (2006, 2009) and Fraga et al. (2007). Unfortunately these studies did not adopt a uniform nomenclature for the lineages (see Rossi et al. 2016 for a detailed review) and did not cover the entire species range, that includes both the Eastern and Western Atlantic coasts and the Eastern Pacific coast (Froese and Pauly 2016). Durand et al. (2012) and Durand and Borsa (2015) showed that these lineages are part of a *Mugil curema* species complex which includes *M. incilis* Hancock, 1830 and *M. thoburni* (Jordan & Starks, 1896), and at least four “*M. curema*” mitochondrial lineages, considered as cryptic species. The first lineage is distributed along the Atlantic coast of the Americas and retains the name of *M. curema*, as the type locality of the original *M. curema* is Bahia, Brazil; the second lineage is present along the Atlantic African coasts and is indicated as *Mugil* sp. M. The third lineage, indicated as *Mugil* sp. N, is present in Venezuela and has recently been formally described as a new species, named *M. margaritae* Menezes, Nirchio, Oliveira & Siccha-Ramirez, 2015 (Menezes et al. 2015). The fourth lineage is distributed along the Pacific coast of the Americas, from the USA to Ecuador, and is indicated as *Mugil* sp. O.

Cytotaxonomy has been proven to be a powerful tool in revealing different lineages/species within Mugilidae. For example, the presence of different cytogenetic features (Nirchio et al. 2003) provided the basis for the identification of an undescribed species, *M. rubrioculus* Harrison, Nirchio, Oliveira, Ron & Gaviria, 2007 (Harrison et al. 2007), as well as the first hints about the existence of cryptic species among mullets, which, until then, had been reported under the name of *M. curema* (Nirchio et al. 2005). Although only two of the four mitochondrial lineages of “*M. curema*” have been cytogenetically investigated to date, they have been found to differ from each other in diploid number and chromosome formula, as well as differing from all the other mugilids investigated to date (see Rossi et al. 2016 for a review). *M. curema* *sensu strictu* shows a karyotype composed of 2n = 28 chromosomes (LeGrande and Fitzsimons 1976, Nirchio et al. 2005) and *M. margaritae* shows a karyotype composed of 2n = 24 chromosomes (Nirchio and Chequea 1998, Nirchio et al. 2005).

This paper reports the cytogenetic analysis of samples of the white mullet collected in Ecuador (Pacific Ocean); according to Durand and Borsa (2015), it is reasonable to assume that they belong to the *Mugil* sp. O. The aim of the study is to describe the karyotype and the cytogenetic features of this *Mugil* sp. and to detect specific cytotaxonomic markers that could be useful for its identification. To verify that all the analyzed specimens belong to the *Mugil* sp. O, sequences of the mitochondrial cytochrome oxidase I (COI) gene were also produced and compared to those previously obtained by Durand et al. (2012) and Durand and Borsa (2015).

## Materials and methods

Seventeen juvenile specimens (undetermined sex), morphologically classified as white mullet (*Mugil curema*) according to Harrison (1995), were caught by cast net at Puerto Hualtaco, at the border between Ecuador and Perú (3°26’S; 80°13’W), and transported alive to the laboratory. The fishes were sacrificed with an overdose of benzocaine (250 mg/l), following the guidelines of the AVMA (2013). Small pieces of muscle and cephalic kidneys were removed from all specimens, and nine individuals preserved in 70% ethanol were deposited as voucher specimens (Table 1).

Total genomic DNA was extracted from muscle according to Aljanabi and Martínez (1997).

**Table 1.** GenBank accession number (A.N.), sampling areas and references of the *Mugil* sp. COI sequences used in phylogenetic analyses.

Individual (Voucher number)	A.N.	Sampling area	Reference
0102 (UTMACH0102)	KU504271	Ecuador	Present paper
0103 (UTMACH0103)	KU504271	Ecuador	Present paper
104	KU504271	Ecuador	Present paper
105	KU504271	Ecuador	Present paper
119	KU504272	Ecuador	Present paper
120	KU504271	Ecuador	Present paper
121	KU504271	Ecuador	Present paper
122	KU504271	Ecuador	Present paper
123	KU504271	Ecuador	Present paper
124	KU504271	Ecuador	Present paper
76104 (LBP 76104)	KU504271	Ecuador	Present paper
76105 (LBP 76105)	KU504271	Ecuador	Present paper
76107 (LBP 76107)	KU504271	Ecuador	Present paper
76129 (LBP 76129)	KU504271	Ecuador	Present paper
76130 (LBP 76129)	KU504271	Ecuador	Present paper
76131(LBP 76131)	KU504271	Ecuador	Present paper
76132 (LBP 76132)	KU504271	Ecuador	Present paper
415	JQ060604	El Salvador	Durand et al. 2012
426	JQ060600	El Salvador	Durand et al. 2012
429	JQ060601	El Salvador	Durand et al. 2012
430	JQ060602	El Salvador	Durand et al. 2012
432	JQ060603	El Salvador	Durand et al. 2012
293	JQ060573	Western Panama	Durand et al. 2012
294	JQ060574	Western Panama	Durand et al. 2012
413	JQ060592	Perù	Durand et al. 2012
420	JQ060595	Ecuador	Durand et al. 2012
423	JQ060597	Western Mexico	Durand et al. 2012
425	JQ060599	Western Mexico	Durand et al. 2012
406	JQ060588	Western Mexico	Durand et al. 2012
422	JQ060596	Western Mexico	Durand et al. 2012
396	JQ060580	Togo	Durand et al. 2012
397	JQ060581	Togo	Durand et al. 2012
390	JQ060575	Senegal	Durand et al. 2012
391	JQ060576	Senegal	Durand et al. 2012
392	JQ060577	Senegal	Durand et al. 2012
393	JQ060578	Benin	Durand et al. 2012
394	JQ060579	Benin	Durand et al. 2012
399	JQ060582	Venezuela	Durand et al. 2012
400	JQ060583	Venezuela	Durand et al. 2012
401	JQ060584	Venezuela	Durand et al. 2012
403	JQ060585	Venezuela	Durand et al. 2012
414	JQ060593	Venezuela	Durand et al. 2012
408	JQ060590	Brazil	Durand et al. 2012
411	JQ060591	Guadeloupe	Durand et al. 2012
419	JQ060594	Belize	Durand et al. 2012
404	JQ060586	Eastern USA	Durand et al. 2012
407	JQ060589	Eastern Mexico	Durand et al. 2012
417	JQ060605	Uruguay	Durand et al. 2012
418	JQ060606	Uruguay	Durand et al. 2012
405	JQ060587	Honduras	Durand et al. 2012
6435	JX559534	Galapagos Is.	Durand et al. 2012
6445	JX559535	Galapagos Is.	Durand et al. 2012
299	JQ060609	French Guyana	Durand et al. 2012
302	JQ060608	French Guyana	Durand et al. 2012
780	HQ285928	Venezuela	Hett et al. 2011
782	HQ285929	Venezuela	Hett et al. 2011
785	HQ285930	Venezuela	Hett et al. 2011
786	HQ285931	Venezuela	Hett et al. 2011
788	HQ285927	Venezuela	Hett et al. 2011

LBP: Laboratório de Biologia e Genética de Peixes, UNESP, Botucatu (São Paulo State, Brazil); UTMACH: Laboratorio de Acuicultura, Universidad Técnica de Machala, Ecuador.

A 546 base-pair (bp) fragment of the mitochondrial cytochrome oxidase subunit I gene (COI) was amplified by PCR using primers FishF1 and FishR2 (Ward et al. 2005) and the procedures reported in Milana et al. (2011). The obtained sequences were aligned using the program MEGA5 (Tamura et al. 2011) and submitted to the GenBank database (https://www.ncbi.nlm.nih.gov/Genbank) under accession numbers KU504271–KU504272 (see Table 1 for details). BLAST (Basic Local Alignment Search Tool) software was used for similarity searching of the COI sequences in GenBank.

Tree reconstructions were conducted using neighbor-joining (NJ), maximum-likelihood (ML) and Bayesian inference (BI) analyses. The NJ and ML analyses (1000 bootstrap replicates) were performed using MEGA5 and PhyML v3.0 (Guindonet al. 2010), respectively. The Bayesian analyses were carried out as implemented in MrBayes v3.1.2 (Huelsenbeck and Ronquist 2001); two independent runs of four Markov chains each for 10^6^ generations were performed. Modeltest v3.7 (Posada and Crandall 1998) and MrModeltest v2.3 (Nylander 2008) were used to select the evolutionary models for the ML and the BI analyses, respectively, according to the Akaike information criterion. All 37 COI sequences of *Mugil* sp. obtained by Durand et al. (2012) and Durand and Borsa (2015), and five COI sequences of *M. incilis*, previously obtained from Venezuelan specimens by our research group (Hett et al. 2011), were included in the phylogenetic analyses (Table 1).

Cell suspensions were obtained from the cephalic kidney, following the procedure reported by Nirchio and Oliveira (2006). Nucleolus organizer regions (NORs) were identified by silver (Ag) nitrate staining (Howell and Black 1980), and C-banding patterns were obtained following the protocol described by Sumner (1972).

Fluorescence in situ hybridization (FISH) was accomplished according to Pinkel et al. (1986). (TTAGGG)n, major (18S rDNA) and minor (5S rDNA) ribosomal probes were amplified by a polymerase chain reaction (PCR) from the genomic DNA of *Eigenmannia* sp. 2, using primers available from the literature (Ijdo et al. 1991, White et al. 1990, Pendás et al. 1994, respectively). The 18S rDNA sequences were labelled during PCR with Digoxigenin-11-dUTP; the 5S rDNA and (TTAGGG)n probes were labelled with biotin-16-dUTP. The detection of hybridization signals was performed using conjugated avidin-fluorescein (FITC) for the 18S rDNA probe and anti-digoxigenin-rhodamine for the 5S rDNA and (TTAGGG)n probes. The chromosomes were counterstained with 4',6-diamidino-2-phenylindole (DAPI) and propidium iodide.

The mitotic figures were photographed using an Olympus BX61 photomicroscope equipped with the appropriate selective filters for FISH and with a DP70 digital camera. The images were digitally edited with Adobe Photoshop CS6 Extended.

## Results

**Figure 1.**
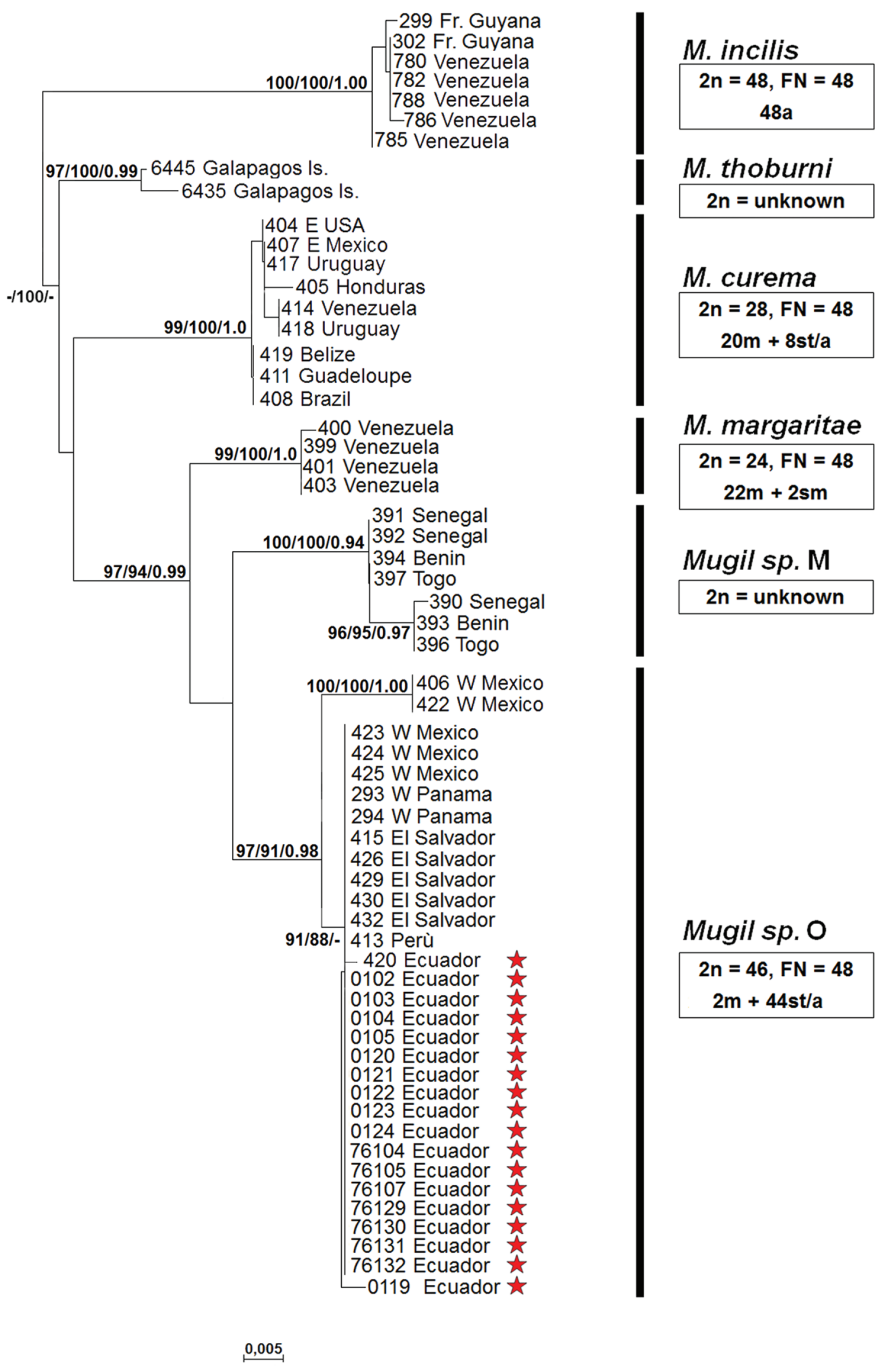
Neighbor-joining tree based on COI sequences. At each node, bootstrap values > 70% (NJ and ML) and posterior probabilities > 0.9 (BI) are shown. Stars indicate sequences obtained in this study; the remaining sequences are from Durand et al. (2012), Durand and Borsa (2015) and from Hett et al. (2011) (see Table 1). For each lineage, the karyotype (2n), the fundamental number (FN) and the chromosome formula are indicated. m: metacentric chromosomes; sm: submetacentric chromosomes; st/a: subtelocentric/acrocentric chromosomes.

Similarity searching of the obtained COI sequences in the GenBank database, using the BLAST function, provided 99.6–100% similarity with those obtained by Durand et al. (2012) and Durand and Borsa (2015) for the Pacific white mullet, i.e., the *Mugil* sp. O. These data were confirmed by the phylogenetic tree topology, obtained by NJ, ML and BI analyses (Fig. 1): all the sequences of white mullets from Ecuador collected in this study clustered within the Pacific *Mugil* sp. O.

**Figure 2.**
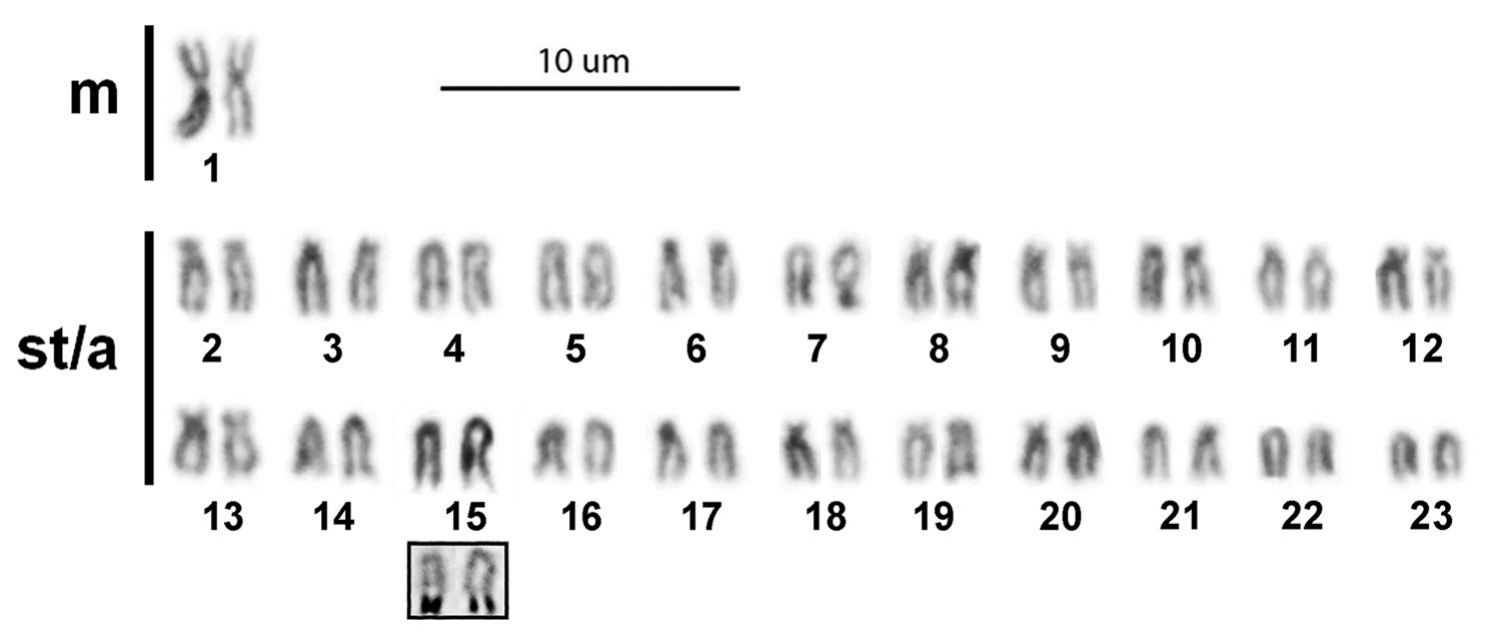
Conventional Giemsa-stained karyotype of the Pacific white mullet. In the inset, the acrocentric chromosome pair n. 15 sequentially Ag-stained; m: metacentric chromosomes; st/a: subtelocentric/acrocentric chromosomes. Scale bar: 10 μm.

In all the individuals, the karyotype is composed of 46 chromosomes, 2 metacentric and 44 subtelocentric/acrocentric, with a fundamental number (FN) of 48 (Fig. 2). The metacentric chromosome pair number 1 was clearly identifiable, whereas the homologues belonging to the subtelocentric and acrocentric series could not be unequivocally identified, due to their uniformly decreasing size. The only exception is the acrocentric chromosome pair classified as number 15 because its homologues show a more or less pronounced terminal achromatic region that is positively stained with AgNO_3_ (Fig. 2, inset).

**Figure 3.**
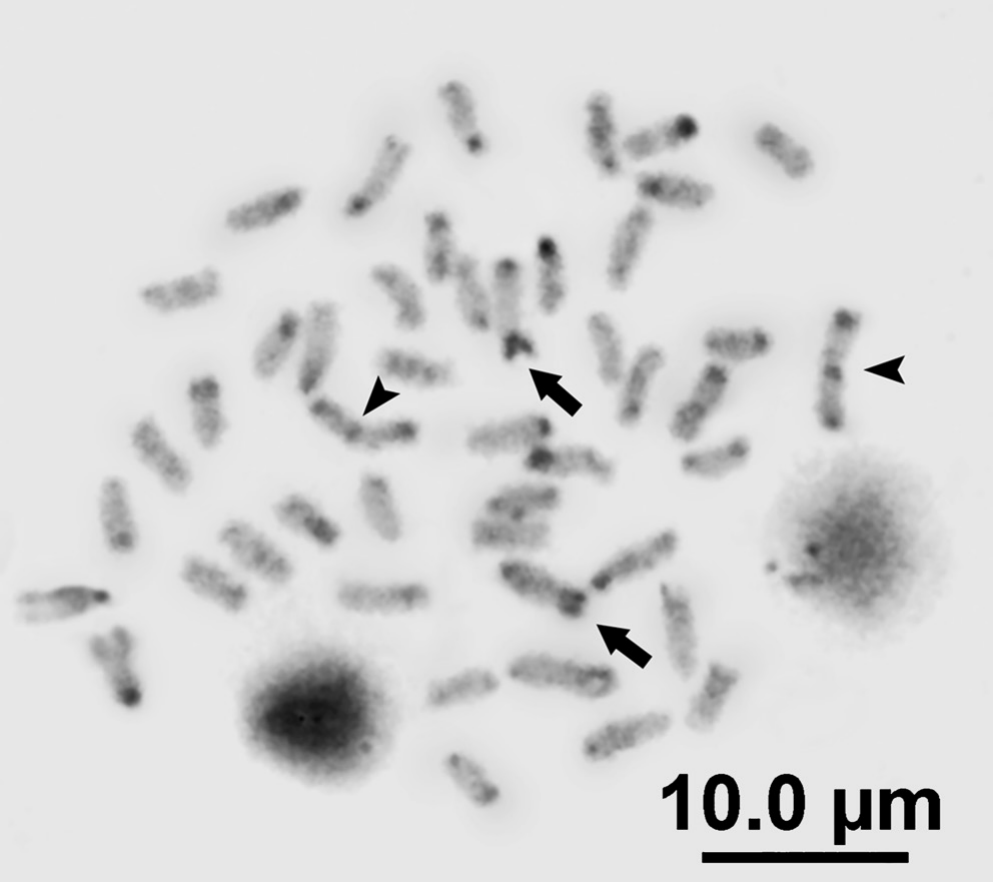
Somatic C-banded metaphases of the Pacific white mullets. Arrowheads indicate chromosome pair number one. Arrows indicate terminal heterochromatic blocks on chromosome pair 15. Scale bar: 10 μm.

C-banding (Fig. 3) revealed the presence of constitutive heterochromatin at the centromeres of most chromosomes and at the telomeres of eight of them. The metacentric chromosome pair number 1 shows C-positive blocks both in the centromeric and in the terminal location; the acrocentric chromosome pair number 15 shows conspicuous heterochromatic blocks in the terminal region.

**Figure 4.**
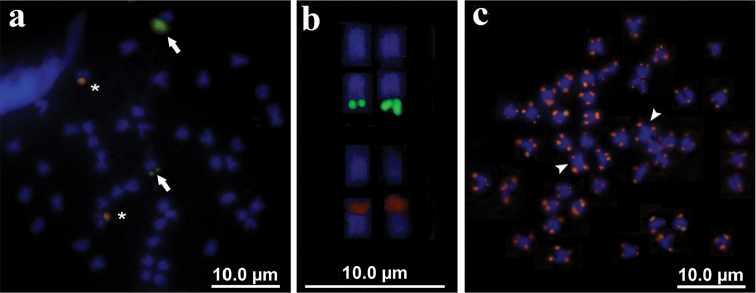
Somatic metaphases of the Pacific white mullet showing positive sites after FISH (**a**) with 18S rDNA (arrows) and 5S rDNA (asterisks) probes and (**c**) with telomeric repeats. Arrowheads indicate chromosome pair number one. In (**b**) enlargement of selected samples of chromosome pairs 15 and 20, after DAPI staining and FISH with rDNA probes, showing 18S (above) and 5S (below) positive sites, respectively. Scale bar: 10 μm.

Dual FISH (Fig. 4a, b) revealed that the 18S rDNA probe yielded two hybridization signals on the same location detected by silver staining on chromosome pair number 15, whereas the 5S rDNA probes hybridized on one smaller medium-sized subtelo/acrocentric chromosome pair (likely number 20) proximal to the centromere.

Mapping of the (TTAGGG)n telomeric repeats showed the presence of positive signals on both telomeres of all chromosomes. No additional, interstitial or centromeric (TTAGGG)n positive signals were detected (Fig. 4c), even on metacentric chromosome pair number 1.

## Discussion

Most of the approximately 20 species of Mugilidae cytogenetically investigated so far (see Rossi et al. 2016 for a review) show a conservative 48 uniarmed (subtelo-, acrocentric chromosomes) karyotype, as well as a conserved FN = 48. Even among the 15 cryptic species identified within the *Mugil cephalus* species complex (Durand and Borsa 2015), the six cytogenetically investigated lineages share not only the chromosome formula but also the major cytogenetic features (see Rossi et al. 2016 for references). The only exceptions to this picture of 2n = 48 are represented by *Liza* (currently *Planiliza) abu* (Heckel, 1843) (Değer et al. 2013) and the two “*M. curema*” lineages so far investigated (LeGrande and Fitzsimons 1976, Nirchio and Cequea 1998, Nirchio et al. 2005). *Planiliza abu* (Değer et al. 2013), which has a limited Asian distribution, shows a karyotype characterized by 2 large metacentric and 46 subtelo-, acrocentric chromosomes, a diploid number of 48 and FN = 50; a pericentric inversion is invoked to interpret the origin of the metacentric chromosome pair. The two cytogenetically known cytotypes of “*M. curema*”, i.e., *M. curema* *sensu strictu* and *M. margaritae*, instead, are both characterized by FN = 48 and by a massive presence of biarmed chromosomes, likely derived from extensive Robertsonian centric fusions of subtelo- and acrocentric chromosomes. The *M. curema* karyotype is composed of 20 metacentric, 4 subtelo- and 4 acrocentric chromosomes, while the *M. margaritae* karyotype is composed of 22 metacentric and 2 submetacentric chromosomes.

The specimens analyzed in this study, molecularly assigned to the Pacific *Mugil* sp. O (Durand and Borsa 2015), show a still-undescribed karyotype in the family, i.e., a diploid number of 46 chromosomes, two of which are metacentrics and 44 of which are subtelo-acrocentrics. Most of the chromosomes of this karyotype are uniarmed, as in the other species belonging to the genus *Mugil*, as well as in different genera of Mugilidae (see Rossi et al. 2016). Nonetheless, the presence of biarmed chromosomes, of a reduced diploid number and of a conserved FN, which are shared with *M. curema* *sensu strictu* and *M. margaritae*, suggests that this karyotype originated by a limited number of centric fusions, i.e., only two uniarmed chromosome pairs were involved. These data confirm that a diploid number different from 48 characterizes all, and so far exclusively, the three investigated “*M. curema*” mitochondrial lineages, with a number of fusions covering the entire range of possibilities (Fig. 1). In *M. margaritae*, all uniarmed chromosomes underwent fusion, in *M. curema* most uniarmed chromosomes underwent fusion, and in the *Mugil* sp. lineage O, only two pairs. At present, it is not possible to discriminate whether the acquisition of the cytogenetic features and/or heterochromatin sequences that promote centric fusions occurred when the ancestor of the *M. curema* species complex split from the other *Mugil* species. Thus, it is not possible to ascertain whether these features were lost in some lineages, or, alternatively, were not acquired at that stage so that not all the molecular lineages were involved. In any case, as the lack of additional telomeric sequences is usually interpreted as a stabilizing factor for fusions (Slijepcevic 1998), the absence of telomeric sequences in a pericentromeric or interstitial position in all the cytogenetically studied “*M. curema*” lineages suggests that Robertsonian fusions are irreversible.

The *Mugil* sp. O described in this study shows the presence of NORs on a single chromosome pair, as well as minor ribosomal genes carried by a single chromosome pair. These features are common to most of the mugilids, including all the *Mugil* species (Rossi et al. 2016). Nevertheless, their location appears to be variable in different species/lineages of the *M. curema* species complex and does not allow any inference on the direction of chromosomal changes within the species complex.

Further analyses are required to draw a comprehensive picture of the chromosomal evolution within the *M. curema* species complex. Data on the karyotype of *M. thoburni* and of the white mullet *Mugil* sp. M from the East Atlantic (Durand and Borsa 2015) are still missing, as well as data on the molecular analysis of the satellite DNA of the whole complex. It is worth noting that in the phylogenetic trees, the node separating “*M. curema*” lineages from *Mugil incilis* was not resolved (Fig. 4, Durand et al. 2012, Durand and Borsa 2015), and the latter species shows a karyotype (Hett et al. 2011) that is the closest to the “typical” all uniarmed mullet karyotype from which, presumably, the “*M. curema*” Robertsonian karyotypes derived. In a very recent paper (Xia et al. 2016) based both on molecular and diagnostic morphological characters, *M. incilis* appears to be the sister species to “*Mugil curema*” lineages, and *M. thoburni* is external to them. Unfortunately, only two of the “*Mugil curema*” lineages (Durand and Borsa 2015) were included in the analysis.

Data, although preliminary, strongly suggest that each of the “*Mugil curema*” lineages within the species complex has its own karyotype. This evidence, and the absence of intermediate karyotypes in the geographic area where different lineages/cytotypes are in sympatry, supports Durand and Borsa’s hypothesis (2015) that chromosomal differences probably prevent interbreeding and indicate the actual reproductive isolation of cryptic species. In this context, a morphological analysis is now needed to assign a species name to the here-examined Pacific *Mugil* sp. O and possibly to the remaining allopatric East Atlantic *Mugil* sp. M.

Finally, it needs to be verified whether the karyotype observed in the specimens from Ecuador is also shared by specimens belonging to the *Mugil* sp. O. from other sampling sites along the American Pacific coast. In particular, a karyotypic analysis is needed for the western Mexican coast, because in the phylogenetic trees two individuals from this region are grouped in a subcluster that is highly divergent from the one that includes the remaining Pacific specimens.
